# Understanding How, Why and for Whom Link Work Interventions Promote Access in Community Healthcare Settings in the United Kingdom: A Realist Review

**DOI:** 10.1111/hex.70090

**Published:** 2024-11-06

**Authors:** Rebecca Golby, Fiona Lobban, Louise Laverty, Kyriakos Velemis, Vishal R. Aggarwal, Katherine Berry, Abby Morris, Emma Elliott, Rebecca Harris, Al Ross, Carolyn A. Chew‐Graham, Miranda Budd, Linda McGowan, David Shiers, Neil Caton, Chris Lodge, Paul French, Robert Griffiths, Jasper Palmier‐Claus

**Affiliations:** ^1^ Lancashire & South Cumbria NHS Foundation Trust Preston Lancashire UK; ^2^ The Spectrum Centre for Mental Health Research Lancaster University Lancaster UK; ^3^ Division of Psychology & Mental Health University of Manchester Manchester UK; ^4^ Greater Manchester Mental Health NHS Foundation Trust Manchester UK; ^5^ School of Dentistry University of Leeds Leeds UK; ^6^ Institute of Population Health, University of Liverpool Liverpool UK; ^7^ School of Health Science and Wellbeing, Staffordshire University Stafford UK; ^8^ School of Medicine Keele University Keele UK; ^9^ School of Healthcare University of Leeds Leeds UK; ^10^ School of Psychology Manchester Metropolitan University Manchester UK; ^11^ Pennine Care NHS Foundation Trust Ashton‐under‐Lyn UK

**Keywords:** community health, healthcare, inequalities, link worker

## Abstract

**Introduction:**

Inequity in access to healthcare in the United Kingdom can have a profound impact on people's ability to manage their health problems. Link work interventions attempt to overcome the socioeconomic and structural barriers that perpetuate health inequalities. Link workers are typically staff members without professional clinical qualifications who support patients to bridge the gap between services. However, little is currently known about how and why link work interventions might be effective. This realist review attempts to understand the contexts and resultant mechanisms by which link work interventions affect access to community healthcare services.

**Methods:**

The authors completed a systematic search of empirical literature in Embase, CINAHL, Medline, PsychInfo and SocIndex, as well as grey literature and CLUSTER searches. Context, mechanism and outcome (CMO) configurations were generated iteratively in consultation with an expert panel and grouped into theory areas.

**Results:**

Thirty‐one eligible manuscripts were identified, resulting in nine CMO configurations within three theory areas. These pertained to adequate time in time‐pressured systems; the importance of link workers being embedded across multiple systems; and emotional and practical support for link workers.

**Conclusion:**

Although link work interventions are increasingly utilised across community healthcare settings, the contexts in which they operate vary considerably, triggering a range of mechanisms. The findings suggest that careful matching of resources to patient need and complexity is important. It affords link workers the time to develop relationships with patients, embed themselves in local communities and referring teams, and develop knowledge of local challenges.

**Patient or Public Contribution:**

The team included people with lived experience of mental health conditions and a carer who were involved at all stages of the review.

## 1 Introduction

There is stark inequity in access to healthcare in the United Kingdom. One of the core principles of the National Health Service (NHS) is equal access to equal needs [1]. However, there is ample evidence that this goal is not always met with the availability of medical care inversely associated with the needs of the populations served [2]. Evidence points to multiple barriers to accessing adequate, appropriate, available, and timely healthcare for disadvantaged populations [3, 4, 5]. Those most in need are likely to delay seeking treatment [6], and a socioeconomic gradient exists in waiting times for services [7]. This is particularly important, as the most deprived are more likely to suffer ill health earlier and for longer period, resulting in gaps in life expectancy [8, 9, 10]. There are concerns that existing inequity in access in the United Kingdom is worsening because of fiscal austerity measures and the COVID‐19 pandemic [11].

The aim of link work interventions is typically to help individuals or families overcome barriers to help‐seeking that widen inequalities in health and well‐being. Link workers are traditionally lay or non‐clinical staff who provide practical support to bridge the gap between patients and clinical systems, services and community organisations. There are many terms for link workers, including ‘social prescribers’ or ‘bridging link workers’. Link work can be differentiated from other healthcare initiatives promoting access to services, such as referrals and signposting, due to the hands‐on nature of the work, often involving multiple contacts and a period of engagement [12]. The link worker role is sometimes akin to that of a support worker, without professional training or formal clinical qualifications, but focuses on navigating services. Link work has been used across primary [13] and secondary [14] care services to facilitate access to a varied range of clinical teams, community groups and peer support. They can sit alone within purpose‐developed services and initiatives or within broader social prescribing schemes. However, despite the varied nature of the link worker role, the objective is often the same: to support marginalised and disadvantaged populations to receive support that they would not otherwise receive.

There is emerging evidence that link work interventions can be effective in supporting access to services in currently under‐served populations, such as people living in areas of multiple deprivation or those living with complex physical and mental health difficulties. For example, it has been effectively used to facilitate dental appointments in families susceptible to poor oral health [15, 16] and diabetes screenings in marginalised inner‐city communities [17]. Despite its promise to improve outcomes, it is also apparent that the link worker role can be complex and challenging with high emotional strain and burnout for practitioners [18], and that there exist multiple systemic barriers to delivery [19]. Little is currently known about the role that contextual factors play in determining the success of link work interventions in facilitating access, and the mechanisms by which they might operate across different service settings. Understanding such processes may enable more effective and appropriate deployment of link work interventions. This is important as one of the aims of the NHS Long Term Plan is that over 900,000 people are able to be referred to a link worker by 2023/24 [20].

Realist reviews seek to make sense of complex interventions, offered across a variety of different contexts [21, 22]. They attempt to understand contexts that trigger mechanisms underlying how interventions ‘work’ by answering the question, ‘What works for whom, in what circumstances, how and why?’ [23, p. 6]. Realist reviews are often theory‐generating (rather than testing) and draw on a range of data sources to develop hypotheses or ideas that lead to programme theory development and refinement. Past reviews in this area have taken a broader focus on social prescribing [24] or a narrower focus on the mechanisms that make link work interventions successful in primary care settings [25], rather than exploring community healthcare settings generally. There have been an unprecedented number of publications focusing on link work interventions in the past four years, suggesting that an updated review is timely. The aim of this realist review was to identify the impacts of link work interventions on access to healthcare services and develop a programme theory to understand the underlying mechanisms that create these impacts, and the contextual factors that trigger these mechanisms to occur.

## 2 Methods

This review adheres to RAMESES published guidance for the reporting of realist reviews [26]. The protocol was published online (PROSPERO: CRD42022302709). Programme theories were developed and refined across the following stages.

### 2.1 Refining the Scope of the Review

The scope of the review was refined by the research team (C.L., J.P.‐C., F.L., L.L., R.G., R.H. and V.A.) and informed by the main aims of this study: to identify the impacts of link work on access to healthcare services and to understand the underlying mechanisms and contextual triggers. The focus was on link worker interventions defined as any intervention in which a patient is linked to/from a community healthcare service in the United Kingdom. Context included any relevant characteristics of the patient or organisational set‐up. The broader political and social context was not prioritised as it was recognised that this would be more difficult to change. The mechanism was defined as the response of any relevant stakeholder to the intervention, including the patient, link worker, staff, and so forth. Outcomes focused specifically on service access for patients as this is the main aim of the link worker role.

### 2.2 Development of Initial Programme Theories

Four authors (E.E., J.P.‐C., R.G. and V.A.) conducted background scoping searches of relevant literature using Medline to begin to identify key literature and emerging programme theories. Subsequently, two authors (R.G. and J.P.‐C.) held Individual, semi‐structured meetings with four expert panel members to generate broad initial theories around how link work interventions support bridging between services across a range of contexts [27]. The expert panel (A.R., C.C.‐G., L.M. and M.B.) consisted of clinical academics specialising in primary and secondary care, and dentistry, known to the lead researchers through academic networks. All had expertise in, or knowledge of, link work interventions. The meetings were used to discuss the panel's experience and knowledge of link working, and their perspectives on what makes link working successful, for whom and in what contexts. The authors used set questions and took detailed notes of conversations. The authors then analysed these notes to identify broad theory areas, which were then iteratively refined by the research team, including people with lived experience of mental health difficulties, and used as a framework to categorise context, mechanism and outcome (CMO) configurations.

### 2.3 Identify Relevant Literature

A systematic search of academic literature was conducted in October 2022 using PsychInfo, CINAHL, SocINDEX, Medline and Embase, which was later updated in August 2024. Two groups of search terms were used: Group 1 related to link work interventions, and Group 2 related to community healthcare services (File S1). These initial search terms were based on key definitions and terminology in the literature (e.g., 18 and 28). The definition of link worker was broad and included advocates, health support workers, social prescribers and support workers, where the aim was to support service access.

The authors conducted a concurrent search of the grey literature using Google, Trip, Allcatsrgrey, the National Institute for Health and Care Excellence (NICE) website, NHS England publications, Clinical Trials, ISRCTN registry, and the UK Government publications website. For each database, variations of the term ‘link worker’ were entered to locate documents not available through traditional database searches, but containing information that could support programme theory generation. Grey literature is included and given particular emphasis in realist research as the aim of the review is to extract information that best answers the research question, regardless of where the data are sourced [28]. Where search results were extensive, the authors screened the first 10 pages of the results from grey literature search engines. Following the main search, the authors employed a purposive CLUSTER approach to identify sibling research of the identified literature, which may have been missed in the original searches [29, 30]. Titles of link work initiatives, based on findings from the initial search, were used to identify further reports (i.e., Rotherham Social Prescribing Service [31], Glasgow Deep End Links Worker Programme [32] and Ways to Wellness Social Prescribing [33]) using the same grey literature databases.

#### 2.3.1 Inclusion and Exclusion Criteria

Papers identified by the search were selected for inclusion based on the criteria in Table 1. In line with guidance around realist reviews [27, 34], literature was not excluded based on study design. Sources were required to provide insights into the mechanisms and contexts in which link work interventions help people to navigate or bridge the gap between clinical services. Within the literature, some authors used the terms ‘social prescriber’ and ‘link worker’ interchangeably, whereas others defined the former as a lighter touch intervention with a less hands‐on approach, focusing on referrals and signposting. For this review, it is acknowledged that the social prescriber role is broader, but the authors accepted reports where the described role approximated that of a link worker. On some occasions, the term ‘well‐being coordinator’ was employed instead of link worker, but again the roles were felt to be analogous by the authors.

**Table 1:** Inclusion and exclusion criteria.

People with a health condition who are linked to/from a community health service (including primary and secondary care)	Link between social care or charity sector services only (i.e., no healthcare setting)
Relate to the United Kingdom	Link between inpatient wards
Focus on a link work intervention	Non‐English language source
	Published before 1990

Two authors (R.G. and E.E.) screened the titles and abstracts of the identified empirical papers before reviewing the full manuscripts against the inclusion and exclusion criteria. Following this, the lead author screened the reference lists and citing articles of relevant literature to identify any further eligible research. For grey literature, in the absence of an abstract, two authors (R.G. and K.V.) screened whole reports and searched for references to link work interventions. Included sources were required to provide insights into the mechanisms and contexts in which link work interventions help people to navigate or bridge the gap between clinical services. This process involved reviewing the literature against the published standards of relevance, richness and rigour to ensure that the data were relevant to the topic area, contextually and conceptually rich, adding depth of understanding and meaning to theory development, and rigorous in its methodology, with a sense of coherence and transparency apparent in its theory development [27, 34, 35]. Grey literature with no reported methodology was assessed for rigour based on the trustworthiness of the source and the credibility of findings [35]. Research with low relevance, richness and rigour, particularly where there was insufficient information to identify CMOs, was excluded.

#### 2.3.2 Data Extraction

Data extraction focused on using abductive and retroductive reasoning to identify CMO configurations to develop programme theories relevant to each broad programme theory area. Each paper was read in depth, and any data relevant to understanding any aspect of a whole or part of a CMO configuration were extracted and coded into the most relevant programme theory area. This was done using Microsoft Excel and allowed multiple coders to contribute to the process.

#### 2.3.3 Analysis and Synthesis Processes

J.P.C., R.G. and F.L. completed the analysis, meeting regularly to discuss findings and identify underlying and recurring CMO configurations (demi‐regularities [36]). The authors then reviewed the configurations and clustered them into groups of theory areas for reporting. Within the analysis, there was particular emphasis on key contextual factors as it was felt that this would facilitate knowledge exchange, highlighting the things that services can act upon to enhance the effectiveness of link worker outcomes in improving access to services.

### 2.4 Use of Formal Theory

The authors attempted to identify how formal theory had been applied to link work interventions and the triggering mechanisms in specific contexts. It was hoped that they could facilitate further in‐depth understanding of the underlying mechanisms by which link worker interventions can improve access. Formal theories were identified by reviewing the literature for mention of theories or models that explained how a link work intervention might work. We considered the relevance of these theories and applied them to our own emerging understanding of CMOs and resultant programme theories.

### 2.5 The Voice of People With Lived Experience

The research team included two people with lived experience of mental health difficulties and a carer. They were involved in all aspects of the review, including the conceptualisation, funding acquisition, analysis and write‐up. The lived‐experience voice was central to creating and understanding emerging theory areas and CMOs.

## 3 Results

### 3.1 Initial Programme Theory Generation

Expert interviews and scoping searches identified preliminary theory areas, which were developed, expanded and refined through the identification of empirical and grey literature. Initial broad theory areas included ideas around the importance of engagement with communities, training and supervision needs and effective information sharing between parts of the system.

### 3.2 Refinement and Development of Programme Theory

**Figure 1:**
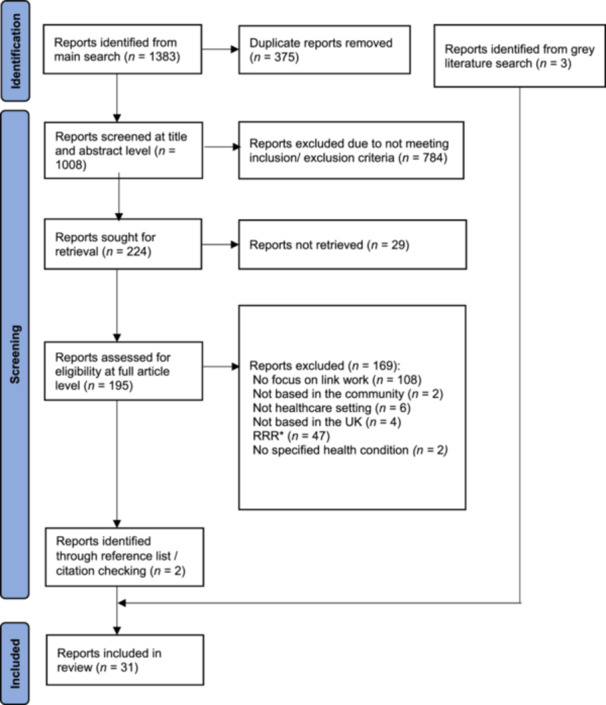
Flow chart showing screening of reports. RRR = relevance, richness and rigour.

Electronic database and grey literature screening yielded 31 reports (Figure 1). Twenty‐eight eligible reports were peer‐reviewed articles and 3 were grey literature [32, 37, 38]. Of these, 28 papers were linked to primary care (including one literature review that included a study involving a Community Mental Health Team), two papers were from secondary care and one paper focused on the Childsmile programme, which links to dental services. Table 2 summarises the included reports.

**Table 2:** Characteristics of included reports.

First author, year	Document type	Study design	Sample/Population	Data collection	Services linked to/from
Aughterson, 2020 [39]	Academic Article	Primary qualitative study	17 GPs	Qualitative interviews	GPs to community groups and activities
Bertotti, 2018 [40]	Academic Article	Primary realist evaluation	17 patients, 3 community organisations, 3 social prescribing coordinators, 2 commissioners and 2 GPs	Qualitative interviews	GPs to community groups and activities
Brunton, 2022 [19]	Academic Article	Primary qualitative study	34 varied staff members around GP surgery	Qualitative interviews	GPs to voluntary, community and social enterprise sector services
Bush, 2014 [17]	Academic Article	Primary quantitative randomised controlled trial	2680 patients	Attendance at screening dates	GPs to diabetes screening clinics
Byng, 2008 [41]	Academic Article	Primary qualitative realist evaluation	21 GPs, 10 varied practice staff and 16 mental health staff	Qualitative interviews	GPs to CMHTs
Calderon‐Larranaga, 2024 [42]	Academic Article	Primary qualitative case study	35 primary care clinicians, link workers and local VCS organisations	Qualitative interviews, ethnographic observations and documentation about the local SP scheme and VCS organisations	GPs to VCS organisations
Chng, 2021 [13]	Academic Article	Primary qualitative process evaluation	121 GP practice staff, and community organisation workers	Qualitative focus groups, email surveys and qualitative interviews	GPs to community resources
Cooper, 2023 [43]	Academic Article	Systematic review	6 papers	Systematic review	Included studies were UK‐based social prescribing services in adults.
Craig, 2008 [44]	Academic Article	Primary mixed methods process evaluation framework	100 patients with tuberculosis diagnosis, and interviews with professionals from 8 care providers	Qualitative interviews and outcome measures	Tuberculosis outpatient clinics to community organisations
Dayson, 2020 [14]	Academic Article	Primary qualitative	2 commissioners, 9 service providers and 9 patients accessing social prescribing	Semi‐structured qualitative interviews	CMHTs to community‐based activities
Department of Health & Social Care, 2023 [37]	Government Report (Grey Literature)	Primary mixed‐methods	165 link workers, 104 green activity providers, 16 stakeholders and 7 health leads	Semi‐structured qualitative interviews, surveys and literature review	Primary care to community green and nature‐based activities
Fixsen, 2020 [45]	Academic Article	Primary critical systems thinking	10 service users, 7 key staff (including 2 link workers), and 8 other stakeholders.	Qualitative interviews	GPs and external organisations to voluntary sector services and community organisations.
Fixsen, 2021 [46]	Academic Article	Primary qualitative interviews	5 managers, 5 GPs, 4 link workers, 3 researchers, 3 community navigators, 2 volunteers and 1 social prescribing advisor	Qualitative Interviews	GPs and community centres to community organisations
Fixsen, 2021b [47]	Academic Article	Primary qualitative interviews	5 managers, 5 GPs, 3 link workers, 3 community navigators, 3 researchers, 2 community volunteers and 1 social prescribing advisor	Qualitative Interviews	GPs and community centres to community organisations
Frostick, 2019 [48]	Academic Article	Primary qualitative	13 link workers	Qualitative interviews and focus groups	Primary care to community organisations
Griffith, 2023 [49]	Academic Article	Primary ethnography	20 link workers	Demographic questionnaire and shadowing of link workers	Primary care to community organisations
Hazeldine, 2021 [50]	Academic Article	Primary qualitative ‘researcher‐in‐residence’ approach	11 link workers, 2 managers and 1 counselling service manager	Qualitative data gained through the lived experience of the ‘Researcher‐in‐Residence’	GPs to voluntary service organisations
Holding, 2020 [51]	Academic Article	Primary qualitative	15 link workers and 9 volunteers.	Qualitative interviews	Community bases to community organisations
Huddart, 2006 [52]	Academic Article	Primary qualitative	23 pupil support staff and integration officers	Qualitative interviews	Primary care and CAMHS staff in a local hospital to community CAMH services
Kellezi, 2019 [53]	Academic Article	Primary mixed‐methods	7 GPs, 9 healthcare providers, 19 service users and 630 patients	Semi‐structured qualitative interviews and longitudinal survey	GPs to relevant third sector groups
Macpherson, 2010 [54]	Academic Article	Primary mixed‐methods	All eligible children in Scotland	Monitoring data collected on dental visits and qualitative work	Link workers (assuming health visitor role) to dental practices
Mercer, 2017 [32]	Report (grey literature)	Primary quasi‐experimental outcome evaluation	84 GP practice staff, 12 patients, 7 GPs, 7 practice managers and 6 link workers	Mixed methods—qualitative interviews and questionnaires	GPs to community services
Moffatt, 2017 [55]	Academic Article	Primary qualitative	30 service users	Semi‐structured qualitative interviews	GPs to community, voluntary and NHS services
Morris, 2022 [56]	Academic Article	Primary qualitative	8 service managers, 5 link workers and 44 clients	Semi‐structured qualitative interviews	GPs to community services
Rhodes, 2021 [18]	Academic Article	Primary qualitative	9 link workers	Semi‐structured qualitative interviews	GPs and other healthcare professionals for community services
Skivington, 2018 [57]	Academic Article	Primary qualitative	6 community links practitioners and 30 representatives of community organisations	Semi‐structured qualitative interviews	GPs to community organisations
Tierney, 2020 [25]	Academic Article	Secondary realist review	118 documents relating to social prescribing	Literature review	Social prescribing services in GPs and community centres to community organisations
White, 2010 [38]	Report (grey literature)	Primary mixed‐methods	12 patients, 6 managers, 3 GPs and 1 nurse practitioner	Routinely collected data, qualitative interviews and a focus group	GPs to community services
Wildman, 2019 [58]	Academic Article	Primary qualitative	24 service users	Semi‐structured qualitative interviews	GPs to community services
Wildman, 2019b [59]	Academic Article	Primary qualitative	15 link workers	Qualitative focus groups and semi‐structured interviews	GPs to community services
Woodall, 2018 [60]	Academic Article	Primary mixed‐methods	436 service users	Semi‐structured qualitative interviews and quantitative questionnaires	Primary care to community services

Abbreviations: CAMH, Child and Adolescent Mental Health Service; CMHT, Community Mental Health Teams; GPs, General Practices; LWP, Links Worker Programme; SP, Social Prescribing; VCS, voluntary and community sector.

During the realist synthesis process, the authors iteratively developed nine CMOs centred around three theory areas: (1) matching resources to complexity in insecure systems, (2) blended embeddedness and (3) supporting those supporting others. The three theory areas represent a refinement of ideas from the initial expert interviews. For example, blended embeddedness was thought to better capture the literature than engagement with communities. Together, the CMOs make up the programme theory. Table 3 displays the CMOs, organised into their respective theory areas, with example quotes supporting each configuration. The CMOs are all interlinked and should not be taken in isolation; it is the combination of these theory areas and CMOs that make link working successful and ultimately lead to better access to services for patients.

**Table 3:** Supporting context‐mechanism‐outcome frameworks for theory areas.

Theory areas/CMOs	Supporting quote(s)
1. Matching resource to complexity in insecure systems.	
1.1. Time in time‐pressured systems. *If link workers are afforded time, this provides opportunities to build relationships and trust between link workers and service users, which better allows them to collaboratively work towards service access*.	*Whilst CMHTs in some areas are able to refer patients to existing primary care‐based schemes, the extent to which this is happening is unclear, and it is arguable that social prescribing services offering a‘lighter*’ *model of provision … would not be able to provide the level of support required by secondary mental health patients*. [14, p. 1]. *As a whole, the users we interviewed appreciated the person‐centred approach of the service and contrasted this with the brief time afforded by GP appointment slots. ‘How often do you get offered an hour's chat about a particular problem with a doctor in the medical centre? You don't, and I have to say that was really quite an incentive’*. [57, p. 8]. *Agencies reported that a major advantage of the post was the additional time, intensive support that the TBLW [Tuberculosis Link Worker] was able to offer patients, sharing of information and raising awareness of the disease.* [58, p. 419].
1.2. Open doors, open now. *If there are few or inadequate local services, this leads to disillusionment in both link workers and patients, who view the service access as an impossible task, causing both to disengage*.	*Unlike most health professionals they can spend more than 5‐10 minutes with patients (first appointments were for one hour), visit them at home if need be, can accompany them to activities and can see them several times over a number of weeks (usually up to six times per client) … None of the patients interviewed thought they would have made the progress they did without the support of the SPHT …‘I just felt relaxed. There's no pressure and it's up to me, they're not pushing me to do anything I don't want to do … she seems understanding*’. [39, p. 28]. *Some link workers had been successful in sourcing sustainable transport for service‐users so they could continue to attend activities at the end of the intervention. However, community transport varied across localities, again reflecting the importance of local infrastructure for the success of social prescribing services.* [41, p. 6]. *[The link worker] said that both of us could go to [the group] the first time, so that she could help me make sure I was comfortable and that I had what I needed to do the class. She spoke to [the instructor] and introduced me to her. I felt a lot happier knowing I had someone I knew to go with me. [lines omitted] If someone had just told me to go, I don't think I would have gone.* [42, p. 5]. *… There's a really bad barrier with the BMI. So, if their BMI doesn't reach a certain level, they can't get onto these weight management programmes, but they're still quite high with their weight, and it's affecting their diabetes risk. So, what I've started to do is try and refer them over to the diabetes centre, for the classes that they do over there, but then they're limited with what they can do as well. … So, unless someone's doing like some healthy eating cooking classes and stuff, but that's not going on right now. So, that is quite hard … but I keep trying [LW 02.5].* [65, p. 4].
1.3. The sustainability of the service offer. *If there is uncertainty in the service offer of link work interventions, link workers feel undervalued, underappreciated and stressed, which leads to staff vacancies and the intervention to break down*.	*When a link worker leaves, they take with them tacit knowledge of local, reliable [service] providers, and relational links. Consequently, improvements made by the service may temporarily decline as a new link worker is installed and has to create positive connections with a range of stakeholders*. [25, p. 7]. *Funders don't like funding existing work, they like funding new stuff. So you're in that dilemma where you know the funder will say* ‘*we're not gonna fund you just to carry on doing what you're doing’. So then people doing the work on the ground, they have to change what they're doing to make it look like a new project and in some cases that changes the whole ethos of the work. And although they might be more successful getting the funding it changes the, you know, the whole dynamic of the delivery.* [38, pp. 31–32]. *[quoting link worker]‘It's a little bit unsettling, that's the truth … when you start bringing organizations in who are tending for contracts and there's a worry about your terms and conditions of contract, if they're going to change your pay and all that type of thing, it's unsettling’*. [43, p. 9]. *‘Everybody's chasing some sort of money. Like the women's centre I'm talking about, they're ‐ they don't know if they're gonna be here in a year's time. They're chasing funding’. [CLP4] [Community links practitioners].*[59, p. 491].
2. Blended embeddedness	
2.1. Co‐location and understanding *If link workers are embedded in referring services, this fosters a sense of teamwork, mutual appreciation and joint working, which streamlines working processes vital to facilitating access*.	*The difference between practices was also apparent in relation to community networking activities. Only in [fully integrated practices] were [link workers] enabled to be proactive and strategic, by, for example, making time each week to interact with staff in community organisations, and facilitating links between community organisations and staff in the practice. These activities were highly valued by the [link workers] in [fully integrated practices].* [13, p. 917]. *To those with investment in practice‐attached schemes, having the link worker ‘embedded within the practice’ with ‘open lines of communication with the GPs in the practice’, was seen as of high importance. Not only did this mean less delay for the patient between GP referral and meeting with the links worker, but also it helped to create a sense of trust for all concerned. These productive discussions about individual clinical cases manifested themselves in different ways in different contexts, and performed a key function of the intervention, in terms of the liaison process.* [43, p. 5]. *This focus on referrals and assessments shaped organisational priorities and made it harder for link workers to engage with complexity and offer the intensive support they felt come clients needed …* ‘that imbalance is a result of structural strategic issues rather than patient need and is incredibly frustrating to me. I'll leave it like that’ (Sam_Focus Group) … Ultimately, link workers increasingly had to secure their own referrals to meet targets … the whole process was time‐consuming and left less time to spend with clients who were increasingly followed‐up o*ver the telephone, rather than face‐to‐face contact.* [60, p. 286].
2.2. Commonality of experience *If link workers are seen as part of the communities they serve, this leads patients to believe in them and their intervention, allowing them to take supported and scaffolded steps towards access*.	*[quoting participant] Individually and collectively they [Wellbeing Coordinators] have worked really hard to get foot hold in their areas, becoming part of forums, neighbourhood networks, health and wellbeing partnerships ….* [49, p. 7]. *Link worker Lucy, had her own experience of ill health … ‘I would send them to someone who was going to give them the time and take them … I feel quite strongly about this’ … she had been instrumental in establishing a local community group that allowed different clients to meet and chat. She often had informal contact with clients, perhaps to remind them of a meeting at the job centre or to turn up to an appointment.* [60, p. 288]. ‘*where I'm living in Tower Hamlets there are lots of takeaways, food, confectionary. … So I see the challenges for other people too [LW 02.3]*’*…* ‘*I'm from a South Asian country, I know …*’ *[LW 02.8]. Yet, practitioners’ opportunities to apply and act upon this relatability varied depending on the context*. [65, p. 4].
2.3. Knowledge of the local services. *If link workers develop knowledge of the local issues and the area, patients and referrals have more faith in their ability to help and are more likely to want to work with them around service access*.	*‘The SPLW service was embedded within both the community and local hospital multi‐disciplinary teams, and SPLWs attended regular meetings for clients who had more complex needs so that they could be referred to relevant services more quickly, to prevent crisis and avoid duplication of care.*[19, p. 7]. *'The link worker's role was therefore considered of fundamental importance in building the relationships with community groups, and enabling effective social prescribing’* (p. 7) ‘*she (the link worker) was a brilliant point of contact just to get plugged into that side of things. Because to be honest, before GP I was completely oblivious to all this stuff'* (p. 9)*. Link workers were also seen as the key ‘bridge’ between the GP and community, where previously GPs were limited by the number of relationships they could build with the different community groups*.’ [47, p. 13[. ***‘**Where service users felt they were referred to a service for activities that did not meet their needs or preferences, naturally they did* ‘*not feel positive about the social prescribing pathway*’*… Themes within studies strongly suggested that service users engagement hinged on whether referrals met their mental health needs or not, as this directly influence the way they would interact with services*. [61, pp. 140–141.
2.4. An ethos of relationship building. *If link work interventions include a focus on relationship building, this will lead to greater trust and confidence in patients, enabling them to engage with the intervention*.	*… having the space and time to explore the context of patient's lives and asking what they thought might help them … It's not,* ‘*You should do this*’*. It's about empowering them to make the decision for their own welfare and wellbeing*. [48, pp 408–409]. *The quality of the relationship between the service user and the link worker was considered essential in six of the included studies. Service users reported* ‘*feeling at ease and relaxed*’ *and* ‘*well‐matched*’ *with their link worker … Having both trust and openness enabled service users to settle into socially prescribed activities and benefit from support that is tailored to their mental health needs.* [61, p. 140]. *Participants favourably contrasted their relationship and interaction with their link worker to their interactions with healthcare professionals, which were often characterised as impersonal and too rushed to properly address the breadth of their social problems.* [62, p. 6]. *The continued link worker relationship was key for Gill [a client receiving the link worker support] in a time of traumatic disruption, and the intervention she received was person‐centred, holistic and consistent.* [63, p. 8].
3. Supporting those supporting others.	
3.1. Training and support to maintain hands‐on focus. *If link workers receive appropriate support and training, they feel confident and clear about their roles, enabling them to become more competent practitioners, which is important for the promoting focused outcomes around access*.	*There is an important coaching element to the role with some patients and those Link Workers with a background in counselling spoke of being tempted to work in greater depth with some patients whilst at the same time realising this was not possible*. [48, p. 409]. *Link workers expressed the need for training so that they could feel confident in those interactions and to protect both themselves and the individuals they were working with.* [64, p. 1848].
3.2. Combating isolation with social support. *If link workers experience isolation, they become burnt out and fatigued, making their roles untenable. Conversely, emotional support can protect link worker well‐being, facilitating their ability to facilitate the link work role*.	*An environment offering supervision or peer support allows anxieties or difficulties associated with the role to be shared and explored. Problems arise when the link worker's capacity and capabilities are overextended, especially if HCPs refer complex cases because (a) they believe the link worker can cope and (b) there is a lack of immediately accessible alternatives (due to long waiting lists for statutory services). The link worker may become so overstretched that they leave their post*. [25, p. 7]. ‘Whilst Link Workers were often passionate about their work, all agreed on the need to be supported in this frontline role where complex and unexpected issues could present themselves without warning … the most important form of support was that of clinical supervision and the safe space it provides to offload and discuss difficult patients and challenging situations …. “we've recently started Clinical Supervision. And I had my first one and it was absolutely amazing … I came out feeling really kind of a weight taken over, a weight that I didn't realise I had had." [48, p. 410].

#### 3.2.1 Theory Area 1: Matching Resources to Complexity in Insecure Systems

The theory area with the most evidence was matching resources with complexity and needs. Resource could take many forms, including the length of sessions, the duration of interventions, the number of practitioners, the size of caseloads and the availability of services in the local area. Patient and community‐level complexity could act as a major barrier to link working, which could be exacerbated by inappropriate resource allocation. If the resource was inappropriately matched, this was felt to contribute to burnout and fatigue in link workers, making them less effective in their roles around supporting service access. Conversely, providing link workers with the capacity and space to work with complexity empowered them to overcome barriers to accessing services. There are three CMOs within this broader theory area.

##### 3.2.1.1 Time in Time‐Pressured Systems

*CMO: If link workers are afforded time, this provides opportunities to build relationships and trust between link workers and service users, which better allows them to collaboratively work towards service access*.

Time had multiple meanings including the number of sessions, the length of sessions and the duration of the intervention, which needed to be appropriate for and tailored to the linked population. It was felt that link workers were able to spend significantly more time with patients compared to other healthcare professionals, which was often seen as a major strength of these interventions. In addition to time availability, efficient time use was also seen as important with more effective and experienced link workers appropriately pacing interventions to meet the needs of patients while achieving the desired outcomes around access. Appropriate time allocation allowed link workers to build relationships and rapport with patients and better understand them as individuals. This in turn helped patients to trust their link workers, giving them confidence in their advice and recommendations. Trust was seen as integral when working closely and collaboratively together, especially when encountering challenging and emotionally demanding barriers to access.

*There is the sense, both from patient and [link worker] interviews, that much of the work actually involves one‐to‐one support over a period of time rather than simply linking patients to community resources …. There was something about the un‐conditionality and continuity of support from [link workers] that was valued by patients.*

[33, p. 58]

Inadequate time and capacity to work with patients were also seen as an obstacle to the success of link work interventions, particularly within the context of complexity and systemic pressures. Link work became more challenging when the practitioner felt forced to pick up a large caseload with multiple needs, but without the capacity or time to sufficiently address their presenting issues. Link workers sometimes described how they had to hurry people into services without building in time to support them to facilitate changes. This led to link workers becoming burnt out and exhausted, making it hard for them to meet the needs of their patients.

*An increase in referral rates by follow‐up had created new challenges. Link workers reported tensions between achieving what were viewed as high referral targets and their ability to deliver the holistic, intensive support their clients needed … Link workers felt they lacked the capacity and/or expertise to offer these clients the high‐intensity, specialist support they needed.*

[40, p. 5]

##### 3.2.1.2 Open Doors, Open Now

*CMO: If there are few or inadequate local services, this leads to disillusionment in both link workers and patients, who view the service access as an impossible task, causing both to disengage*.

There was a strong suggestion in the identified reports that the success of link work interventions was contingent on the availability of local services, which were often impacted by local funding limitations and cuts [38, 51, 53]. Access problems included long waiting times, changing service remits, financial barriers and service closures. Although challenges around transport could sometimes be addressed during the intervention, this was sometimes seen as a barrier to progress being maintained once the link work intervention had finished.

*Of particular value was the potential to be engaged with the service for up to 2 years. Due to the long‐term and complex nature of conditions which often fluctuated, participants recognised that a shorter‐term approach would be inadequate …‘It's the kind of thing if you need them, you phone them and they'll get straight back to you. They're there, I know they're there*’ *[patient speaking about link worker].*

[28, p. 6]

Reports highlighted the benefits of having multiple onward referral options so that they were able to tailor their offer to the person's interests and needs [51, 55]. This was particularly true of link work within the context of social prescribing schemes where patients were referred from NHS services to local charities, support groups and societies, which appeared to be instrumental in generating enthusiasm and interest from referrers and patients for the linking process.

*The availability of services varied greatly across localities. For example, a link worker operating in an affluent area in the South of England discussed the abundance of community activities in their local area: [quoting participant]'There's groups for everything, you know. Everything I thought of which would make an idea for a group, there's a group out there ….'*

[41, p. 1540].

##### 3.2.1.3 The Sustainability of the Service Offer

*CMO: If there is uncertainty in the service offer of link work interventions, link workers feel undervalued and underappreciated, which leads to staff vacancies and the intervention to break down*.

Although less supported in the empirical literature, some sources of information raised the possibility that the stability of link worker service provision and investment in link worker interventions determined their success [37, 46]. In the absence of long‐term funding commitments and job security, link workers felt stressed and underappreciated within the navigated systems. Some link workers saw their roles as precarious and voiced concerns that rates or conditions of pay and job roles might alter with changes in contracts or service providers. This was further compounded by uncertainty in career progression and how people felt that they could build on the link worker role. Service‐level uncertainty also appeared to affect referrers' trust in link work interventions, as referrers were reluctant to refer patients due to uncertainty around how long the intervention would be in place, and therefore how much benefit it would provide to patients, resulting in fewer referrals over time.

*In all three schemes, there were uncertainties about future funding of both social prescribing schemes and third‐sector organizations. As one Link Worker explained, ‘We really don't know what that's going to look like ahead, unfortunately, we just don't. And that's a huge, huge worry*’.

[44, p. 675]

#### 3.2.2 Theory Area 2: Blended Embeddedness

A second theory area concerned the idea of blended embeddedness; the need for link workers to be seen to have roots both within referring services and the communities that they served. Within this were ideas around being perceived as a member of the team by referrers and this being integral to building professional trust and respect. However, link workers were also expected to be situated within and belong to the local community, which was important for building a sense of togetherness and understanding with patients. The success of link work interventions was therefore contingent on the link workers' ability to concurrently embed themselves into and navigate these two social spaces. There were four CMOs within this theory area.

##### 3.2.2.1 Co‐Location and Understanding

*CMO: If link workers are embedded in referring services, this fosters a sense of teamwork, mutual appreciation and joint working, which streamlines working processes vital to facilitating access*.

Reports highlighted the importance of placing link workers within referring services, emphasising the need for a perceived and real presence within teams [13, 41, 46, 52]. If link workers could incorporate themselves into services, this elicited good information sharing and communication. It enabled link workers to formally and informally advise and liaise with staff on possible referrals and update them on the work completed. This in turn helped to build a sense of collaboration and togetherness with referring clinicians, which produced greater numbers of appropriate referrals, with clear goals and targets for the link work intervention, resulting in improved outcomes.

*A positive feedback cycle occurred when link workers became integrated into primary care teams, with productive case discussions also leading to increased trust and generating further liaison opportunities … Productive clinical case discussions were reported to be the key to both link worker integration and better outcomes.*

[45, p. 5].

Conversely, if link workers and referrers were seen as outsiders, it could be difficult for them to build relationships and sell the benefits of their intervention. This was particularly true of primary care settings where General Practitioners did not see engagement with link work interventions as a core part of their role [19, 47, 52, 59]. In turn, this could lead to feelings of disconnection and detachment between referrers and link workers, which led to fewer referrals and limited ability to bridge between services. In some instances, link workers who had been unable to develop links with referrers felt despondent and a sense of failure.

*… staff referred to changes in joint working that had a negative impact on the service: ‘Co‐location increases understanding but …recently [the link worker] feels more isolated … is [now] in a room on [their] own’.*

[46, p. 32]

##### 3.2.2.2 Commonality of Experience

*CMO: If link workers are seen as part of the communities they serve, this leads patients to believe in them and their intervention, allowing them to take supported and scaffolded steps towards access*.

Initial expert interviews highlighted the importance of link workers belonging to the same communities as patients, which was then supported by several academic articles across multiple settings [14, 39, 55]. Embeddedness was helped by recruiting link workers from similar backgrounds, who knew the local language, values and culture. It was felt that this commonality of experience afforded link workers greater empathy, understanding and compassion towards their patients. In turn, when this passion was visible, it facilitated trust and confidence in patients, which allowed them to take steps towards accessing services that were previously unavailable, scaffolded and supported by somebody that they knew and trusted.

*These kinds of experience helped Link Workers to sit with whatever was brought to the session and relate to the patient … Empathy and the ability to listen empathetically was widely regarded as an essential skill as were other personal attributes like the ability to be non‐judgemental and persevere to build trust.*

[48, p. 410]

##### 3.2.2.3 Knowledge of the Local Services

*CMO: If link workers develop knowledge of the local challenges and the area, patients and referrals have more faith in their ability to help and are more likely to want to work with them around service access*.

Link workers who were embedded in an area were viewed as having insider and rich knowledge of local systems and services. This afforded them a practical advantage in finding places to refer to, but also gave stakeholders confidence in their ability to facilitate bridging between services. At times, this local knowledge highlighted to referrers the advantages of using the link worker, rather than making referrals and bridging services themselves, contributing to a throughput of referrals and the success of the intervention. There was some acknowledgement that acquiring local knowledge was time consuming and required the researching and scoping out of services, linking to CMO 1.1. During expert panel interview 6, it was discussed that unfortunately, disruption caused by the COVID pandemic was seen to have broken down ‘internal maps’ of services meaning that link workers had to relearn the local context all over again.

*The Wellbeing Coordinators suggested that they had worked consistently to build a presence in various geographical areas, building relationships with a number of different services and organisations and understanding the local offer in communities and neighbourhoods … The approachability, trustworthiness and communication skills of the Coordinator were crucial and often results in individuals feeling valued and listened to … The interpersonal qualities of the Wellbeing Coordinator were raised as a key factor in service users engaging with the social prescribing service.*

[49, p. 7]

##### 3.2.2.4 An Ethos of Relationship Building

*CMO: If link work interventions include a focus on relationship building, this will lead to greater trust, confidence and sense of agency in patients, enabling them to engage with the intervention*.

Multiple reports stressed the importance of relationship building within link work interventions, rather than being entirely task focused. Again, this was closely associated with time allocation (CMO 1.1), but also related to an ethos and directive from management and supervisors on how link workers should conduct their sessions. Furthermore, it was reliant on the link workers' interpersonal effectiveness, experience, and passion for the area. If link workers prioritised and effectively built relationships with patients, learning their stories, backgrounds and presenting problems, this instilled confidence, trust and a sense of agency in patients, which in turn empowered them to engage with the intervention. This had to be a whole team approach to function well.

*Such workers demonstrated genuine passion and drive, fostered through previous community‐based roles, and utilised innovative strategies to address gaps in community infrastructure … In common with other research into the importance of the link worker role in developing relationships, engaging service‐users and ultimately improving outcomes.*

[41, p. 1542]

#### 3.2.3 Theory Area 3: Supporting Those Supporting Others

The third theory area focuses on the importance of effective and standardised training and supervision to help link workers to feel competent, confident and focused in their roles, but that emotional support was equally as important. Link workers were seen as susceptible to emotional burnout, isolation and role drift making it particularly important that they received the right support when they needed it. There are two CMOs within this theory area.

##### 3.2.3.1 Training and Supervision to Maintain Hands‐On Focus

*CMO: If link workers receive appropriate support and training, they feel confident and clear about their roles, enabling them to become more competent practitioners, which is important for promoting focused outcomes around access*.

Supervision and training were seen as integral to the success of link work interventions, which sometimes included behavioural‐change strategies, cognitive behavioural techniques and motivational interviewing. This had the benefit of empowering link workers to be more hands‐on in their roles, elevating interventions from lighter‐touch forms of signposting to engaging and comprehensive support. Supervision was also seen as important for overcoming the multifaceted and complex barriers to access, which required collective thinking and creative problem solving.

*[quoting participant] we're almost like social prescribing but we also would provide intervention … around behavioural change as well. Because the idea being that these might be repeat presenters to their GP. They're not just going to go somewhere because you tell them ….*

[19, p. 4]

Link workers came from a variety of backgrounds, including housing support, debt management, exercise, health coaching and counselling. Although the diversity of experience was considered a strength of the workforce, some reports suggested the risk of *role drift*, where both link workers and referrers were confused about the nature and purpose of the intervention, leading to inappropriate referrals and varied outcomes. Structured training and supervision helped link workers maintain focus in their work leading to better outcomes around access, rather than getting lost in the complexity of their caseloads.

*While this variation in skillset and expertise within teams was highlighted as a positive aspect, it also raised concerns that it could affect the consistency of offer across areas, meaning that signposting and other aspects of the role were undertaken differently by different professionals.*

[19, p. 6]

##### 3.2.3.2 Combating Isolation With Emotional Support

*CMO: If link workers experience isolation, they become burnt out and fatigued, making their roles untenable. Conversely, emotional support can protect link worker well‐being, facilitating their ability to facilitate the link worker role*.

Several reports emphasised the importance of formal and informal emotional support for link workers. It was clear that link work could, at times, be challenging and demanding for the practitioners themselves, affecting their well‐being and mental health. Link workers often operated independently in the community and/or as part of small teams, which could be isolating. This increased the need for regular emotional support from peers, families and supervisors. Frequent informal, empathic conversations were seen as paramount to protecting link workers from fatigue, exhaustion and burn‐out, which could affect their ability to work effectively and therapeutically with patients.

*Where [social prescribers] were unable to find support from another [social prescribers], they felt increased anxiety regarding their ability to fulfil their role and cope emotionally as others’ understanding of the role was perceived to be limited … [social prescribers] working for this organisation all commented that they would find the role untenable without this support.*

[18, pp. 342–343]

### 3.3 Identification and Discussion of Formal Theory

Within the literature, there was scarce use of theory to interpret and understand the contexts and resultant mechanisms in which link work interventions might operate. The most relevant was the application of Social Capital Theory and Patient Activation Theory by Tierney et al. [25] who previously reviewed link work interventions in primary care. The authors outlined how link work interventions can facilitate social capital, which relates to resource from the connections that form between people, and can reduce isolation, generate meaning in life, and act as a conduit for advice. Applying Patient Activation Theory, they suggest that social capital can result in patient activation (i.e., increased confidence, motivation and ability): small successes that build into higher levels of achievement. Our emerging programme theory proposes a similarly important role for relationship building and further elucidates the structural antecedents that allow this to occur (e.g., matching of resource allocation to complexity and need, embeddedness with communities, provision of training and supervision). Although Tierney et al. [25] propose a role for reducing isolation in patients, our programme theory suggests that social embeddedness and tackling isolation in link workers are key factors in determining whether these interventions can promote access for people in under‐served groups.

Mercer et al. [32] created a programme theory of change to underpin the Glasgow Deep End Links Worker Programme. This highlighted the resources and actions available at a patient, practice and community level that could facilitate short‐, medium‐ and long‐term outcomes. One‐to‐one individual support was theorised to lead to skills, information and support acquisition, resulting in better self‐management of health conditions, systems, crises and challenges. This, in turn, was hypothesised to result in better relationships between patients and professionals and enhanced patient well‐being in the long term. The proposed activities at a practice level to promote staff well‐being are consistent with CMO 3.1, which highlights emotional support as a key context. Similarly, the idea of link workers gathering ‘intelligence’ (p. 16) on available resources is akin to knowledge of local services (CMO 2.3). The programme theory also highlights the importance of blended embeddedness calling for integration of link workers into community hubs.

Aughterson, Baxter, and Fancourt [39] mapped qualitative interview data to the capability, opportunity and motivation domains of the COM‐B model to understand barriers and enablers to GPs utilising social prescribing interventions, such as link work. Their data suggested that link work interventions could facilitate relationship building and community integration, enhancing all domains of the model. Two reports [40, 48] mentioned psychological theories, namely social learning theory [61] and attachment theory [62], to explain the mechanisms by which relationships might foster behaviour change; through patients' modelling of link worker behaviour and the creation of secure relationships that allow a person the freedom and confidence to navigate their environment, which are pertinent to CMO 2.4.

## 4 Discussion

The aim of this review was to explore the mechanisms by which link work interventions affect service access in community healthcare settings and the contextual factors that trigger these mechanisms to occur. Expert interviews identified initial programme theories, which were subsequently developed and refined through the identification and analysis of 31 reports. The analysis suggests that link workers require time resources matched to levels of complexity and need, stable service provision, places to link to, and emotional and practical support. Their success is also contingent on link workers’ ability to embed themselves into multiple systems, within the communities they are serving, with the freedom to build relationships and knowledge of the local service offer. These, in turn, can build trust, self‐confidence and understanding of the link work process in both patients and referrers, which appears vital for facilitating positive outcomes around access.

The authors hypothesise that link work interventions must be appropriately resourced and supported to match the complexity and need of the linked population in order to enable access, which should be considered in the face of long‐term fiscal challenges in the NHS. Link workers require time, flexibility and permission to emphasise relationship building with patients. As the NHS adopts short‐term interventionist models of healthcare, there is a risk that longer term enablers of change are deemphasised in policy, service design and commissioning. However, our findings suggest that relationship building should not be overlooked in favour of brief practical solution‐based interventions. Indeed, one framework applied to link work interventions was attachment theory, which suggests that the formation of secure relationships is essential for allowing individuals to explore and navigate their social environments [62]. Indeed, there is some evidence that secure attachment can lead to a better therapeutic alliance between clinicians and patients in health and social care settings and engagement with services [63].

The current analysis highlights the importance of link workers maintaining a dual role as a professional and as a member of the local community, demonstrating shared experience, to build trust and respect over time. Therefore, link workers may have to engage both individuals and their communities, while also managing their role within teams. Wildman et al. [59] refer to the need for ‘well‐networked’ link workers, acknowledging that they are harder to recruit but integral to the successful delivery of these interventions. Other authors have also highlighted the importance of link workers utilising their life experiences in their roles to build relationships [48], which resonates with literature around the potential benefits of lived experience and peer support [64]. However, in this review, the authors failed to identify any research that had formally used peer support workers as link workers.

The programme theory suggests that link work interventions require adequate provision around training and supervision, affording clear direction and focus to avoid role drift, as well as emotional support. Without this, link workers themselves are vulnerable to isolation, anxiety and burn‐out, which are associated with high staff absenteeism and turnover, as well as worse patient safety [65]. One hypothesis was that if link work interventions lack focus, this can lead referrers to disengage from the process, which could have ramifications for their delivery in practice.

Realist reviews can ‘open the black box’ [27] of interventions, illuminating how these work, for who and in what contexts. It is vital that the findings are recognised as theories and subject to the interpretation and experiences of the authors, shaped through reflection and feedback from the expert panel. These theories need further testing and refinement through the evaluation of specific link worker programmes. Most reports identified in this review of link work interventions were published recently, in the past four years, suggesting that this is an emerging and relevant area of investigation. The authors identified little grey literature in this area (*n* = 3) and more might have strengthened the emerging programme theories. Most link worker interventions were situated in primary care. Only two studies explored link work in the context of secondary mental health services [14, 41] and one, albeit large, evaluation of link work in an oral health pathway [54]; the contexts and mechanisms by which link work interventions act on access in these areas may be under‐represented in the current analysis and require further consideration and theorising. All studies were based in the United Kingdom; therefore, the applicability of the theories beyond this context is limited.

## 5 Conclusion

Link working is a complex intervention operating in complex systems. This review has attempted to understand what makes community link working successful in increasing access to services for patients in the United Kingdom. Based on our evidence‐led conclusions, the authors advise that, when developing and designing community link work interventions, consideration is given to the topics raised in this review with particular emphasis on the adequate training and supervision of link workers, who must be embedded in local communities and referring teams. The authors also advise that resource is matched to the local population's needs, affording link workers enough time to develop positive relationships with patients and support them in accessing appropriate services.

## Author Contributions

**Rebecca Golby:** conceptualisation, investigation, writing–original draft, methodology, writing–review and editing, formal analysis, project administration, data curation. **Fiona Lobban:** conceptualisation, investigation, funding acquisition, writing–original draft, methodology, supervision, formal analysis. **Louise Laverty:** formal analysis, conceptualisation, investigation, funding acquisition, writing–original draft, supervision, methodology. **Kyriakos Velemis:** writing–review and editing, methodology, validation, data curation. **Vishal R. Aggarwal** and **Katherine Berry:** conceptualisation, investigation, funding acquisition, writing–review and editing. **Abby Morris:** conceptualisation, investigation, writing–review and editing, methodology, formal analysis. **Emma Elliott:** project administration, data curation, writing–review and editing. **Rebecca Harris:** conceptualisation, investigation, funding acquisition, writing–review and editing, methodology, project administration. **Al Ross** and **Carolyn A. Chew‐Graham:** visualisation, writing–review and editing, validation. **Miranda Budd:** validation, visualisation, writing–review and editing. **Linda McGowan:** validation, visualisation, writing–review and editing. **David Shiers:** conceptualisation, investigation, funding acquisition, writing–review and editing, visualisation, validation. **Neil Caton** and **Chris Lodge:** validation, visualisation, writing–review and editing, conceptualisation, investigation, funding acquisition. **Paul French:** validation, visualisation, writing–review and editing, funding acquisition, conceptualisation. **Robert Griffiths:** conceptualisation, funding acquisition, validation, visualisation, writing–review and editing. **Jasper Palmier‐Claus:** conceptualisation, investigation, funding acquisition, writing–original draft, methodology, validation, visualisation, writing–review and editing, formal analysis, project administration, supervision, resources.

## Ethics Statement

The authors have nothing to report.

## Conflicts of Interest

D.S. is an expert advisor to the NICE Centre for Guidelines; the views expressed are those of the authors and do not reflect those of NICE. The other authors declare no conflicts of interest.

## Supporting information

Supporting information.

## Data Availability

The authors have nothing to report.
