# Supplementary material for: Understanding How, Why and for Whom Link Work Interventions Promote Access in Community Healthcare Settings in the United Kingdom: A Realist Review
Source: Health Expect. 2024 Nov 6;27(6):e70090. doi: 10.1111/hex.70090 (PMC11540931; doi:10.1111/hex.70090)
Supplement: Supplementary file 1 — Supporting information. [file HEX-27-e70090-s001.docx]

Supplementary Table 1. Search terms

| **Search engine** | **Search terms** |
| --- | --- |
| **PsychInfo** | S1 ( DE "Community Mental Health Services" OR DE "Community Counseling" OR DE "Community Services" OR DE "Community Mental Health Services" OR DE "Community Welfare Services" OR DE "Emergency Services" OR DE "Home Care" OR DE "Home Visiting Programs" OR DE "Public Health Services" OR DE "Health Care Delivery" OR DE "Clinical Practice" OR DE "Health Care Access" OR DE "Health Care Costs" OR DE "Health Care Reform" OR DE "Health Care Utilization" OR DE "Managed Care" OR DE "Quality of Care" OR DE "Quality of Services" OR DE "Health Care Services" OR DE "Behavioral Health Services" OR DE "Continuum of Care" OR DE "Electronic Health Services" OR DE "Health Care Delivery" OR DE "Health Screening" OR DE "Hospital Programs" OR DE "Long Term Care" OR DE "Mental Health Services" OR DE "Palliative Care" OR DE "Patient Centered Care" OR DE "Prenatal Care" OR DE "Primary Health Care" OR DE "Mental Health Programs" OR DE "Crisis Intervention Services" OR DE "Deinstitutionalization" OR DE "Home Visiting Programs" OR DE "Hot Line Services" OR DE "Suicide Prevention Centers" OR DE "Mental Health Services" OR DE "Community Mental Health Services" OR DE "School Based Mental Health Services" ) OR TI ( ((health OR clinical OR outreach OR communit*) N3 (service* OR clinic* OR clinics OR team*) ) OR ("out patient*" OR outpatient* OR out-patient*) ) OR AB ( ((health OR clinical OR outreach OR communit*) N3 (service* OR clinic* OR clinics OR team*) ) OR ("out patient*" OR outpatient* OR out-patient*) )  TI ( (link N2 work*) OR (bridg* N2 work*) OR (health* N2 (advoca* OR "support work*" OR supportwork* OR support-work*))) OR AB ( (link N2 work*) OR (bridg* N2 work*) OR (health* N2 (advoca* OR "support work*" OR supportwork* OR support-work*)))  britain or british or “united kingdom” or uk or england or wales or Scotland or "Northern Ireland" or “great britain”  Restricted to 1990 onwards.  Restricted to English language.  Restricted to Human. |
| **CINAHL** | TI ( (link N2 work*) OR (bridg* N2 work*) OR (health* N2 (advoca* OR "support work*" OR supportwork* OR support-work*))) OR AB ( (link N2 work*) OR (bridg* N2 work*) OR (health* N2 (advoca* OR "support work*" OR supportwork* OR support-work*)))  (MH "Health Services+") OR (MH "Community Mental Health Services+") OR (MH "Health Services Accessibility+") OR (MH "Health Services Research+") OR (MH "Community Health Services+") OR (MH "Mental Health Services+") OR (MH "Dental Health Services+") OR (MH "Maternal Health Services+") OR (MH "National Health Programs+") OR (MH "Emergency Medical Services") OR TI ( ((health OR clinical OR outreach OR communit*) N3 (service* OR clinic* OR clinics OR team*) ) OR ("out patient*" OR outpatient* OR out-patient*) ) OR AB ( ((health OR clinical OR outreach OR communit*) N3 (service* OR clinic* OR clinics OR team*) ) OR ("out patient*" OR outpatient* OR out-patient*) )  britain or british or “united kingdom” or uk or england or wales or Scotland or "Northern Ireland" or “great britain”  Restricted to 1990 onwards.  Restricted to English language.  Restricted to Human. |
| **SocINDEX** | (DE "HEALTH services accessibility" OR DE "UNIVERSAL healthcare" OR DE "CLINICAL sociology" OR DE "MENTAL health" OR DE "MENTAL health & social status" OR DE "SOCIAL psychiatry" OR DE "HEALTH" OR DE "HEALTH attitudes" OR DE "HEALTH self-care" OR DE "HEALTH status indicators" OR DE "MEN'S health" OR DE "SOCIAL determinants of health" OR DE "WOMEN'S health") OR TI ( ((health OR clinical OR outreach OR communit*) N3 (service* OR clinic* OR clinics OR team*) ) OR ("out patient*" OR outpatient* OR out-patient*) ) OR AB ( ((health OR clinical OR outreach OR communit*) N3 (service* OR clinic* OR clinics OR team*) ) OR ("out patient*" OR outpatient* OR out-patient*) )  TI ( (link N2 work*) OR (bridg* N2 work*) OR (health* N2 (advoca* OR "support work*" OR supportwork* OR support-work*))) OR AB ( (link N2 work*) OR (bridg* N2 work*) OR (health* N2 (advoca* OR "support work*" OR supportwork* OR support-work*)))  britain or british or “united kingdom” or uk or england or wales or Scotland or "Northern Ireland" or “great britain”  Restricted to 1990 onwards.  Restricted to English language. |
| **Medline** | (MH "Health Services+") OR (MH "Health Services Accessibility+") OR (MH "Preventive Health Services+") OR (MH "Health Services Research+") OR (MH "Mental Health Services+") OR (MH "Community Health Services+") OR (MH "Dental Health Services+") OR (MH "Maternal Health Services+") OR (MH "Health Services Needs and Demand+") OR TI ( ((health OR clinical OR outreach OR communit*) N3 (service* OR clinic* OR clinics OR team*) ) OR ("out patient*" OR outpatient* OR out-patient*) ) OR AB ( ((health OR clinical OR outreach OR communit*) N3 (service* OR clinic* OR clinics OR team*) ) OR ("out patient*" OR outpatient* OR out-patient*) )  TI ( (link N2 work*) OR (bridg* N2 work*) OR (health* N2 (advoca* OR "support work*" OR supportwork* OR support-work*))) OR AB ( (link N2 work*) OR (bridg* N2 work*) OR (health* N2 (advoca* OR "support work*" OR supportwork* OR support-work*)))  britain or british or “united kingdom” or uk or england or wales or Scotland or "Northern Ireland" or “great britain”  1 AND 2 AND 3  Restricted to 1990 onwards.  Restricted to English language.  Restricted to Human. |
| **Embase (Ovid)** | exp health care/ or exp health service/ or exp community care/ or exp mental health/ or ((health or clinical or outreach or communit*) and (service* or clinic* or clinics or team*)).mp. or ("out patient*" or outpatient* or out-patient*).mp.  ((link or bridg*) adj2 work*).mp. or (health* adj2 (advoca* or "support work*" or supportwork* or support-work*)).mp.  (britain or british or "united kingdom" or uk or england or wales or Scotland or "Northern Ireland" or "great britain").mp.  1 AND 2 AND 3  limit 4 to (human and english language and yr="1990 - 2022") |
